# Does abortion reduce self-esteem and life satisfaction?

**DOI:** 10.1007/s11136-014-0687-7

**Published:** 2014-04-17

**Authors:** M. A. Biggs, Ushma D. Upadhyay, Julia R. Steinberg, Diana G. Foster

**Affiliations:** 1 Advancing New Standards in Reproductive Health, University of California, San Francisco, San Francisco, CA USA; 2 Department of Psychiatry, University of California, San Francisco, San Francisco, CA USA; 3 1330 Broadway, Suite 1100, Oakland, CA 94612 USA

**Keywords:** Abortion, Self-esteem, Life satisfaction, Well-being

## Abstract

**Purpose:**

This study aims to assess the effects of obtaining an abortion versus being denied an abortion on self-esteem and life satisfaction.

**Methods:**

We present the first 2.5 years of a 5-year longitudinal telephone-interview study that follows 956 women who sought an abortion from 30 facilities across the USA. We examine the self-esteem and life satisfaction trajectories of women who sought and received abortions just under the facility’s gestational age limit, of women who sought and received abortions in their first trimester of pregnancy, and of women who sought abortions just beyond the facility gestational limit and were denied an abortion. We use adjusted mixed effects linear regression analyses to assess whether the trajectories of women who sought and obtained an abortion differ from those who were denied one.

**Results:**

Women denied an abortion initially reported lower self-esteem and life satisfaction than women who sought and obtained an abortion. For all study groups, except those who obtained first trimester abortions, self-esteem and life satisfaction improved over time. The initially lower levels of self-esteem and life satisfaction among women denied an abortion improved more rapidly reaching similar levels as those obtaining abortions at 6 months to one year after abortion seeking. For women obtaining first trimester abortions, initially higher levels of life satisfaction remained steady over time.

**Conclusions:**

There is no evidence that abortion harms women’s self-esteem or life satisfaction in the short term.

## Introduction

Life satisfaction and self-esteem are indicators of well-being [1, 2]. According to Abraham Maslow’s hierarchy of five basic needs, self-esteem is considered necessary before an individual can reach the highest level of self-actualization or realize her full potential [3]. Low self-esteem has been found to lead to adverse physical and mental health conditions, delinquency, and lower socioeconomic status [4, 5]. Life satisfaction is strongly associated with health and longevity [6], and a greater likelihood of becoming and staying married, having kids, and having a happy and stable vocational life [7].

While there has been substantial interest in understanding the effects of abortion on women’s mental health and well-being, the body of literature examining whether abortion is related to self-esteem and life satisfaction is limited. To the extent that the literature addresses these topics, they are considered secondary to other mental health outcomes, particularly depressive, anxiety, and substance use disorders [8–11]. The notion that abortion lowers women’s self-esteem has been the basis, in part, for legislation to restrict abortion access. In 2007, Supreme Court Justice Anthony Kennedy wrote in the Gonzales V. Carhart case: “While we find no reliable data to measure the phenomenon, it seems unexceptionable to conclude some women come to regret their choice to abort…. Severe depression and loss of esteem can follow” [12].

The few studies examining abortion and self-esteem outcomes have methodological limitations making it difficult to establish the relationship between abortion and self-esteem. Some studies fail to control for known confounders and preexisting mental health conditions [13–15] and/or are limited by their lack of a comparison group [16]. One longitudinal study followed women just before a first trimester abortion to 2 years after obtaining the abortion and found that women’s self-esteem improved over time [16]. However, lacking any comparison group, this study was unable to assess whether the self-esteem trajectories of women undergoing an abortion differ when compared to other women who had unintended pregnancies. Others use comparison groups that do not account for the factors that lead women to terminate a pregnancy, for example, by comparing women having an abortion to pregnant women who carry to term irrespective of their pregnancy intentions [15, 17–19]. Two studies that compared women who had abortions to women who gave birth or had miscarriages did not find differences in post-pregnancy self-esteem [17, 18]. One used 5 years of longitudinal data from the National Longitudinal Study of Adolescent Health and found that adolescents who had an abortion did not have subsequent lower self-esteem compared to those who had a pregnancy that ended in miscarriage or delivery [18]. Similarly, another study used data from the National Comorbidity Survey and found that abortion versus delivery of a first pregnancy was not associated with self-esteem at the time of interview [17]. In contrast, two studies that compared women who had abortions to other women found that those having abortions had subsequent higher self-esteem [15, 19], although in one, the association was not significant once demographic factors were included in analyses [15].

We found only one study to examine the effect of abortion on life satisfaction. This study was conducted in Norway and followed 39 women who experienced a miscarriage and 70 women who had an induced abortion, for 5 years, and found no group differences in their perceived quality of life [20]. For both groups, perceptions of quality of life improved over time. This study did not consider women’s pregnancy intentions when selecting its comparison group.

The disregard for women’s pregnancy intentions in the literature examining the effects of abortion on women’s well-being is problematic. In order to understand whether abortion influences the self-esteem or life satisfaction trajectories of women, one should compare them to a group of women who also experience unintended pregnancies. Thus, one important comparison group is women who are seeking, but are denied abortion.

Here, we focus on positive psychological outcomes including self-esteem and life satisfaction. This is the first study to prospectively assess whether women’s self-esteem and life satisfaction trajectories differ up to 2.5 years after abortion seeking, by comparing women who have an abortion to women who were denied an abortion and have to carry their pregnancy to term.

## Methods

### Study design overview

The *Turnaway Study*, a prospective longitudinal study of women seeking abortions was designed to test the notion that abortion harms women by improving on many of the methodological shortcomings found in the existing literature. Some of the strengths found in this study include its longitudinal design, and use of women denied a wanted abortion as a comparison group. Using data from the *Turnaway Study*, this paper aims to assess whether self-esteem and life satisfaction changes following an abortion and whether the trajectories of women undergoing an abortion differ from those denied an abortion. The study design included three distinct groups: (1) women denied an abortion because they were up to 3 weeks over the facility’s pregnancy gestational age limit (*Turnaways*), (2) women who received an abortion and were up to 2 weeks under the facility’s gestational age limit (*Near Limits*), and (3) women who received a first trimester abortion (*First Trimesters*). Study details have been described elsewhere [21–23].

### Recruitment

From 2008 to 2010, we recruited women seeking abortion care at 30 facilities in 21 states throughout the USA. Facilities were identified using the National Abortion Federation membership directory and by referral. Sites were selected based on their gestational age limits to perform an abortion procedure, where each facility had the latest gestational limit of any facility within 150 miles. Gestational age limits ranged from 10 weeks to the end of the second trimester. Facilities performed over 2,000 abortions a year on average [24].

Participants were recruited in a 1:2:1 ratio for each study group (*Turnaways*, *Near Limits*, and *First Trimesters*, respectively). Study eligibility criteria included meeting gestational age criteria described above, being English or Spanish speaking, 15 years of age or older, and having a pregnancy without a fetal diagnosis or demise. At each site, a designated point person was trained to oversee and conduct recruitment activities, screen for eligibility, and inform potential participants about the study. Participants who expressed willingness to learn more about the study were then connected to *Turnaway* study researchers by telephone. During the recruitment call, research staff explained the study in greater detail, screened for eligibility and obtained informed consent. The first baseline telephone interview took place 8 days after either receiving or being denied an abortion. Baseline interviews lasted approximately 40 minutes. The study is ongoing, with follow-up phone interviews being conducted every 6 months for 5 years. Here, we present the first 6 waves of data which span the first two and half years of follow-up interviews.

All interviewers were female, fluent in English and/or Spanish, and experienced in reproductive health research and interviewing techniques. The interviewer training covered general interviewing guidelines, handling sensitive issues, confidentiality, data collection protocols, question-by-question reviews of both English and Spanish versions of the interview guide, role playing, and record keeping. During the data collection period, research staff worked closely with the interviewers to ensure data quality. The interviewers simultaneously collected and entered data into a password protected, computer database using Computer-Assisted Survey Execution System (CASES). Women were mailed a $50 gift card for a major retail store after completing each interview. Written and oral consent was obtained from all participants. This study was approved by the University of California, San Francisco, Committee on Human Research.

### Measures

The structured interview guide included questions on participant socio-demographic characteristics, experiences becoming pregnant, pregnancy planning, and their health and well-being. The interview guide and study protocols were first pilot tested among 64 women receiving or being denied an abortion at a local abortion facility.

#### Outcome variables

We considered two ordinal outcome variables. Self-esteem was a one-item measure of global self-esteem, a validated measure designed as an alternative to the Rosenberg Self-Esteem Scale [25]. Life satisfaction was one item selected from the five-item Satisfaction with Life Scale (SWLS) [26]. For both measures, participants were asked to “describe how well the following statements describe how they have been feeling in the last 7 days, including today.” These two statements included “Felt high self-esteem?” and “Felt satisfied with your life.” Participants responded to these two items on a five-point scale, ranging from one (*not at all*) to five (*extremely*).

#### Predictor variables

Study group was the main predictor variable and included four groups. *Turnaways* were separated into two groups (1) the “*Turnaway No Birth*” group included those who obtained an abortion elsewhere or had a miscarriage, and, (2) the “*Turnaway Birth*” group included those who went on to have a live birth; the additional two groups were (3) *Near Limits* which included those who were just under the facility’s gestational limit and had an abortion, and (4) *First Trimesters* which included those who had abortions in their first trimester. The 15 Turnaway women who placed their babies for adoption are included in the *Turnaway Birth* group. Study group, time (months since seeking abortion), and study group by time interactions served as our predictor variables.

#### Potential covariates

Control variables consisted of baseline demographic and other characteristics known to be associated with the two outcome variables. Specifically, they included age, race/ethnicity (White, Black, Hispanic/Latina and other), highest level of education (less than high school, high school or GED, Associates/Technical degree or some college, and college degree or higher), marital status, employment status (part/full time versus not employed), parity, history of child abuse/neglect, history of depression or anxiety, illicit drug use, and problem alcohol use (either drinking first thing in the morning or not being able to remember what happened after a night of drinking) prior to pregnancy recognition.

### Statistical analysis

Baseline differences between *Near Limits* and the other three study groups were assessed using mixed effects regression analyses to account for clustering by site and included linear mixed effects regression for continuous variables and logistic mixed effects regression for dichotomous variables (Table 1). For multinomial variables such as race, education, marital status, and parity, a post-estimation test was performed to test for overall differences on the variable of interest by study group.

**Table 1:** Baseline participant characteristics by study group

	Total	Study Groups			
		Near limit abortion	Turnaway births^a^	Turnaway no births^b^	First trimester abortion^c^
	*n* = 877	*n* = 413	*n* = 161	*n* = 49	*n* = 254
Age in years, mean	24.9	24.9	23.4**	24.5	25.9*
Race/ethnicity (%)					*
White	33	32	25	43	39
Black	32	32	34	29	32
Hispanic/Latina	22	21	29	12	21
Other	13	15	13	16	8
Highest level of education (%)					
<High school	19	18	25	18	16
High school or GED	33	34	34	27	31
Associates/technical degree/some college	40	35	35	47	42
College	8	6	6	8	11
Employed (%)	54	54	40**	49	63*
Marital status (%)					
Single	79	80	84	78	76
Married	9	8	10	6	11
Divorced/widowed	12	12	6	16	13
Gestational age in weeks, mean	17.0	19.9	23.4***	19.1***	7.8***
Parity (%)			*		
Nulliparous	38	34	47	41	38
One birth	28	32	24	29	25
Two or more births	34	34	29	31	37
History of child abuse or neglect (%)	26	26	26	12*	28
Prior depressive or anxiety disorder diagnosis (%)	25	23	21	29	30
Any illicit drug use prior to pregnancy (%)	14	13	14	8	18
Problem alcohol use prior to pregnancy (%)	6	4	7	10	7

^a^Turnaway births compared to near limit abortion group

^b^Turnaway no births compared to near limit abortion group

^c^First trimester abortion comparison group compared to near limit abortion group

* *p* < 0.05; ** *p* < 0.01; *** *p* < 0.001

The main statistical analyses include longitudinal mixed effects linear regressions [27] to assess whether self-esteem and life satisfaction trajectories differ between women denied an abortion and women who had an abortion, and whether levels of self-esteem and life satisfaction differ at the initial interview. Since the majority (90 %) of abortions in the USA occur in the first trimester of pregnancy [28], comparisons between the *Near Limits* and the *First Trimesters* served to assess whether the experiences of women seeking later abortions differ from the typical experience of women having abortions in the USA. *Near Limits* serve as the reference group to allow multiple comparisons between groups.

Adjusted models include study group and time (months) as the primary independent variables, and control for baseline covariates that could potentially confound the relationship between study group and self-esteem and life satisfaction outcomes. All analyses adjust for clustering by including random intercepts for facility and subject. We tested whether adding group by time interactions, random slopes for individuals, and fixed quadratic terms for time, improved the model fit using likelihood ratio tests, and included these when appropriate. We conducted a separate analysis to test whether the available sample at 2½ years was biased with regards to our outcome variables by assessing whether there were significant differences in baseline self-esteem or life satisfaction among those participating and those subsequently lost to follow-up at each follow-up wave. We fit all models using STATA 12.

## Results

### Participant characteristics

Overall, 37.5 % of eligible women agreed to complete semi-annual telephone interviews for a period of 5 years, with no differential participation by study group. A total of 956 women completed a baseline interview 8 days after seeking an abortion. One facility was excluded (*n* = 76) from all analyses because 95 % of women initially denied an abortion obtained one elsewhere and therefore did not have an adequate sample of *Turnaways*. Three *Near Limit* and *First Trimester* women were excluded because they chose not to have an abortion after agreeing to participate in the study, leaving a final sample of 877 participants. In our final analyses, *Turnaways* included the *Turnaway Births* (*n* = 161) and *Turnaway No Births* (*n* = 49) groups (which included 44 women who received an abortion elsewhere and 5 who had a miscarriage). The *Near Limits* included 413 women, and the *First Trimesters* included 254 women. Ninety-two percent of the 877 participants who completed the first interview also completed the six-month follow-up, and 72 % (*n* = 634) were retained at the 2.5 year interview. There were no significant differences in baseline self-esteem or life satisfaction among those participating and those subsequently lost to follow-up, at any of the five follow-up interview waves.

Table 1 summarizes the baseline participant characteristics by study group. Groups were similar with regards to highest level of education completed and marital status. By design, gestational age at recruitment differed by study group. When compared to *Near Limits*, *Turnaway Births* were more likely to be younger, unemployed, and nulliparous. *Turnaway No Births* were less likely to report a history of child abuse or neglect when compared to *Near Limits* and more likely to report problem alcohol use before discovering pregnancy. The *First Trimesters* were older, more likely to be employed, and more likely to be White than *Near Limits*.

The results of the adjusted mixed effects regression models comparing trajectories of self-esteem and life satisfaction by study group are presented in Table 2. We included group by time interactions, random slopes for individuals, and quadratic terms for months because likelihood ratio tests suggested that these improved the model fit (*p* < 0.05). In the adjusted models without group by time interactions, significant coefficients for months indicated that overall, self-esteem (*β* = 0.02, 95 % CI 0.01, 0.02) and life satisfaction (*β* = 0.01, 95 % CI 0.008, 0.014) improved over time (not shown).

**Table 2:** Longitudinal mixed effects regression analyses predicting self-esteem and life satisfaction trajectories by study group

Predictor variables	Self-esteem			Life satisfaction		
	Coef.	95 % CI		Coef.	95 % CI	
Study group						
Near limit abortion (reference)						
Turnaway births	−0.28*	−0.52	−0.04	−0.14	−0.37	0.08
Turnaway no births	−0.33	−0.71	0.07	−0.38*	−0.75	−0.01
First trimester abortion	0.11	−0.10	0.31	0.19*	0.0004	0.39
Months	0.04***	0.03	0.05	0.03***	0.01	0.04
Turnaway births × months	0.02	−0.01	0.05	0.02	−0.01	0.05
Turnaway no births × months	0.02	−0.03	0.07	0.03	−0.01	0.08
First trimester abortion × months	−0.02	−0.04	0.01	−0.03**	−0.05	−0.01
Months^2^	−0.001***	−0.001	−0.0004	−0.001**	−0.001	−0.0001
Turnaway births × months^2^	−0.0002	−0.001	0.001	−0.0003	−0.001	0.001
Turnaway no births × months^2^	0.0001	−0.001	0.002	−0.001	−0.002	0.001
First trimester abortion × months^2^	0.0005	−0.0003	0.001	0.001*	0.00005	0.001
Control variables						
Age	−0.01	−0.03	0.00	−0.03***	−0.04	−0.01
Race/ethnicity						
White (reference)						
Black	0.38***	0.20	0.56	−0.05	−0.21	0.11
Hispanic/Latina	0.01	−0.19	0.20	−0.01	−0.19	0.17
Other	0.24*	0.01	0.47	0.01	−0.20	0.22
Highest level of education						
<High school (reference)						
High school or GED	0.23*	0.03	0.43	0.24*	0.06	0.42
Associates/technical degree/some college	0.45****	0.25	0.65	0.36***	0.18	0.54
College	0.36*	0.04	0.68	0.29	0.003	0.58
Employed	0.21**	0.06	0.35	0.08	−0.05	0.21
Marital status						
Single (reference)						
Married	0.00	−0.25	0.24	0.10	−0.12	0.32
Divorced/widowed	−0.13	−0.36	0.10	−0.10	−0.31	0.10
Parity						
Nulliparous (reference)						
One birth	−0.04	−0.22	0.14	−0.15	−0.31	0.01
Two or more births	0.05	−0.15	0.25	−0.06	−0.24	0.11
Child/abuse neglect	−0.16	−0.32	0.01	−0.24**	−0.38	−0.09
Previous anxiety of depression diagnosis	−0.41***	−0.58	−0.24	−0.38***	−0.53	−0.23
Any drug use before discovering pregnancy	−0.12	−0.32	0.08	−0.34***	−0.52	−0.16
Problem alcohol use	0.00	−0.30	0.30	−0.02	−0.29	0.24

*Coef*. regression coefficient, CI 95 % confidence interval

* p < 0.05, ** p < 0.01, *** p < 0.001

### Self-esteem trajectories

**Fig. 1:**
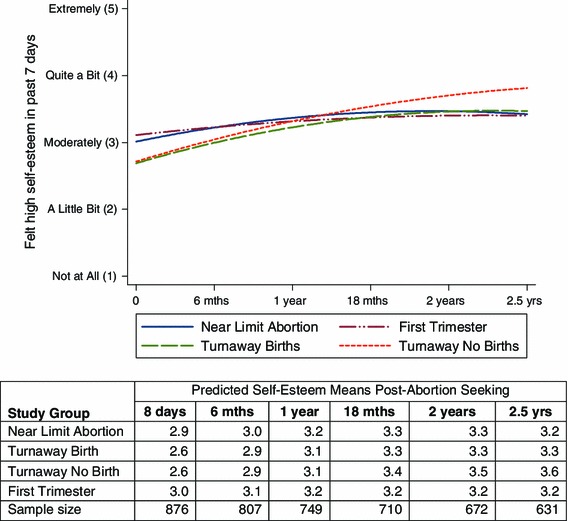
Self-esteem trajectories 2.5 years following abortion seeking by study group

In the self-esteem model including the group by time interaction terms, we found that the *Turnaway Birth* group had significantly lower baseline self-esteem (*β* = −0.28, 95 % CI −0.52, −0.04) than the *Near Limits* (Table 2). The lack of significant group by time interactions in this model indicates that the self-esteem trajectories of *Near Limits* do not differ significantly from the other study groups. Seen graphically (Fig. 1), self-esteem appears initially lower for both Turnaway groups when compared to the *Near Limits* and *First Trimesters* and the lines for all groups converge after about 1 year

### Life satisfaction trajectories

**Fig. 2:**
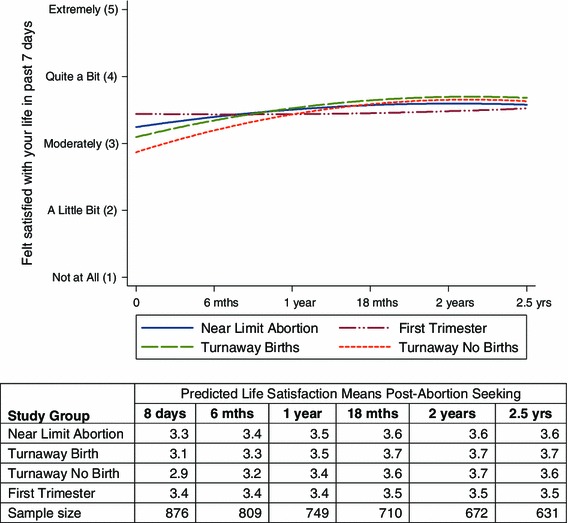
Life satisfaction trajectories 2.5 years following abortion seeking by study group

In the life satisfaction model including the group by time interaction terms, we found that at baseline, when compared to *Near Limits*, the *Turnaway No Births* had lower life satisfaction (*β* = −0.38, 95 % CI −0.75, −0.01) and the *First Trimesters* had higher life satisfaction (*β* = 0.19, 95 % CI 0.0004, 0.39, Table 2). The significant First Trimester × Months and First Trimester × Months square interactions in the model fitting life satisfaction indicate that the life satisfaction trajectories differ between the *Near Limits* and *First Trimesters* (Table 2; Fig. 2). As seen in Fig. 2, the initial levels of life satisfaction among *First Trimesters* remain steady over time, whereas life satisfaction starts out lower and gradually improves over time for all other groups; all study group differences in life satisfaction appear to disappear by 1 year.

## Discussion

This study demonstrates that there is no increased risk of low self-esteem or life dissatisfaction following an abortion relative to being denied one. Our study design is well suited to assess Justice Kennedy’s statement that loss of self-esteem follows an abortion and finds no evidence to support this assumption. Instead, over the course of the study, self-esteem improved or remained unchanged among women who had an abortion. In fact, these findings suggest that the alternative to having an abortion—being denied an abortion—is more harmful to women’s feelings of self-worth and well-being, at least at the outset, than having an abortion. Women unable to get an abortion were more likely to have lower self-esteem and life satisfaction a week after being denied an abortion, when compared to women who terminated their pregnancies. Yet, even for these women who had unwanted births, self-esteem and life satisfaction eventually improved, reaching the same levels of women who underwent the abortion procedure by 6 months–1 year after seeking an abortion.

At baseline assessment, women in all four study groups had lower than average self-esteem when compared to a national sample of women, using the same validated measure, which returns to average after about 1 year [29]. The initial lower self-esteem and life satisfaction felt by women is possibly a consequence of having an unintended pregnancy, and the conditions which make a pregnancy unwanted–lacking the financial resources or “right partner” or simply not wanting a child at the time of becoming pregnant—which led to the decision to terminate the pregnancy [30]. The process of seeking an abortion—finding a facility, finding time for the procedure, travel and procedure costs, and opposition from others— may also have affected self-esteem and life satisfaction [22]. Being denied a wanted abortion may have further reduced or maintained women’s lower than average self-esteem and satisfaction with life. The initial decrement and recovery of life satisfaction have also been observed among breast cancer survivors [31, 32]. The gradual increases in self-esteem observed in this study parallels that of other studies where self-esteem is reported to gradually increase after adolescence, peaking in late mid-life [29].

This study improves on existing research because it was designed to assess whether abortion negatively affects women’s well-being using an appropriate comparison group of women who is equally motivated to terminate their pregnancies, but do not receive the abortion. This comparison group is appropriate because it considers the factors that lead women to decide to have an abortion, such as pregnancy intentions and life circumstances. Furthermore, by following women for over 2 years after seeking abortion, we were able to map the self-esteem and life satisfaction trajectories of multiple groups of women faced with an unintended pregnancy. Findings from this study offer a methodologically robust contribution to the literature on the effects of abortion on women’s well-being.

One limitation in the study design is that life satisfaction is assessed with a single-item measure, whose validity and reliability have not been tested. This one item was taken from a five-item measure that has been found to be robust and a valid measure of life satisfaction. The single item used in this study has been shown to have the highest item-total correlations of all the five items, with correlations of 0.71 and above [33]. Thus, while not validated, our measure does capture an important component of satisfaction with life. Another study limitation is that for survey research standards, our participation rate of less than 40 % is low. However, given that women were approached at a very inconvenient time (at abortion seeking), and asked to participate in 11 lengthy interviews over 5 years, concerning a very stigmatized and personal topic (their abortion), it is not surprising that our response rate is not comparable to cross-sectional studies. Nonetheless, self-esteem and life satisfaction outcomes may have differed from those who consented and those who did not consent to participate in this study. In previous multilevel analyses, facility characteristics were associated with participation [23]; participation was higher in facilities located in more liberal political settings than those seeking abortion in conservative areas. Thus, those who may have faced the most stigma for seeking abortion in their communities may not have been adequately captured by this study. When we compare our participation rate to that of other prospective studies of this type, we find that the response rates are similar [34, 35] and our retention rate is somewhat higher [16]. Losing <6 % from wave to wave, our participant retention rate is high, strengthening the validity of our findings. Furthermore, while the sample is over representative of women seeking abortions at later gestational ages, the sample demographics are consistent with those of nationally representative samples of women seeking abortion in the USA suggesting that these results are generalizable [36, 37]. By over-representing women seeking abortions later in pregnancy, we were able to assess any changes in well-being among this less studied population. To our knowledge, this is the first study to prospectively assess women’s self-esteem and life satisfaction among a relatively large sample of women seeking abortion beyond the first trimester.

While an abortion may be an emotionally significant event in a woman’s life, there is no evidence or other reason to believe that it causes harm to self-esteem or life satisfaction in the short or long term. These findings are consistent with previous findings from the Turnaway study that shortly following their abortion, the majority of women expressed feelings of relief about their abortion and felt the abortion was the right decision for them [38]. Women denied abortion felt more regret and anger and less relief and happiness than those who obtained their abortion. The current study indicates that abortion in and of itself does not cause women to be dissatisfied with their lives nor hinder their self-esteem. The experience of unintended pregnancy or the circumstances that lead women wanting to terminate the pregnancy are more likely the factors associated with lower self-esteem and life satisfaction around the time of seeking abortion. Efforts to support women’s emotional well-being should focus on these and other factors known to impact women’s self-esteem and life satisfaction.
